# Case Report: Clinical, molecular, and functional characterization of autoimmune lymphoproliferative syndrome—a family study with a multimodal diagnosis

**DOI:** 10.3389/fped.2025.1639749

**Published:** 2025-07-18

**Authors:** Bruna Cândido Guido, Ricardo Camargo, Caroliny Victoria dos Santos Silva, Anna Carolina Silva Dias, Robéria Mendonça de Pontes, Agenor de Castro Moreira dos Santos Júnior, Raquel Alves Toscano, Fabíola Scancetti Tavares, Alexandre de Albuquerque Antunes, Karina Mescouto de Melo

**Affiliations:** ^1^ Laboratório de Pesquisa Translacional, Hospital da Criança de Brasília José Alencar, Brasília, Distrito Federal, Brazil; ^2^ Allergy and Immunology Unit, Hospital da Criança de Brasília José Alencar, Brasília, Distrito Federal, Brazil; ^3^ Hematology Department, Sabin Medicina Diagnóstica, Brasília, Brazil

**Keywords:** autoimmune lymphoproliferative syndrome (ALPS), diagnosis, immunophenotyping, *FAS* variant, *CASP10* variant, apoptosis defects, functional assay

## Abstract

Autoimmune Lymphoproliferative Syndrome (ALPS) is a rare immunological disorder caused by defective apoptosis, commonly due to pathogenic variants in the *FAS* gene. We report a comprehensive clinical, immunophenotypic, molecular, and functional evaluation of nine members of a consanguineous Brazilian family harboring the pathogenic *FAS* (NM_000043.6:c.748C > T) variant. The index case, an 11-year-old boy, presented with recurrent cytopenias, splenomegaly, and increased double-negative T cells. Genetic analysis identified additional variants in *CASP10* (NM_032977.4:c.1202_1208del), and *LRBA* (NM_001364905.1:c.2450-7C > T), evidencing a complex genotype. Functional assays confirmed different levels of impaired FAS-mediated apoptosis in some affected individuals. Among nine family members studied, four out them met clinical and molecular criteria for ALPS, demonstrating incomplete penetrance and variable phenotype. All affected individuals share the same variants in *FAS* and *CASP10*, yet their clinical presentations differ significantly. Clinical manifestations and elevated double-negative T cells were observed exclusively in male individuals. Notably, a female family member harboring both *FAS* and *CASP10* variants remained asymptomatic, supporting previous findings of incomplete penetrance and suggesting that sex-related factors—possibly including hormonal influences—may modulate clinical expression in ALPS. Introduction of sirolimus therapy led to sustained remission in the index case. This study report a successful integration of multimodal diagnostic strategy for accurate identification and management of ALPS, and it highlights the potential role of targeted therapies in improving outcomes.

## 1 Introduction

Autoimmune Lymphoproliferative Syndrome (ALPS) is a rare immunological disorder characterized by defective apoptotic mechanisms that disrupt lymphocyte homeostasis. Clinical manifestations include multilineage cytopenias, lymphadenopathy, hepatomegaly, splenomegaly as well as autoimmune diseases and increased risk of lymphoma (1, 2). Its prevalence remains uncertain, although it is estimated that there are several hundred cases globally (3).

Pathogenic variants in the *FAS* gene are the main cause of ALPS. In addition to *FAS*, other variants in apoptosis-related genes such as *FASLG*, *CASP10*, *CASP8*, *FADD* and *NRAS* are described in a small group of patients (4, 5). In this context, the *FAS*:c.748C > T (p.Arg250Ter) variant emerges as a genetic alteration of interest, since it is associated with the clinical severity of ALPS (6–8). This variant results in the replacement of the arginine codon by a premature termination codon, p.Arg250Ter, affecting FAS receptor functioning and leading to cellular resistance to pogrammed death. This resistance is particularly relevant in the pathogenesis of ALPS, since the accumulation of autoreactive lymphocytes translates into an intensification of both autoimmune activity and clinical severity (9). In parallel, although the pathogenicity of some *CASP10* variants remains controversial (10, 11), some studies suggest that they may act as disease modifiers by subtly impairing apoptotic signaling in synergy with other genetic alterations (12).

Patients with ALPS have an expansion of TCRαβ+CD4-CD8- cells (DNT cells), which correlates with dysfunction in the regulation of the immune response. Increased levels of interleukin-10, vitamin B12, and the presence of defective FAS-mediated apoptosis are also frequent and suggest impairment in the programmed cell death mechanism, essential for immune homeostasis (13).

Although *FAS* variants are a well-established cause of defective apoptotic signaling in ALPS, their impact on apoptosis is not always the same. In this study, we observed different levels of apoptotic activity among patients carrying the same *FAS* variants. The phenotypic variability observed among patients can be attributed to genotypic events that affect the expression of other genes involved in apoptosis and in the immune response, which can result in cases ranging from asymptomatic to severe clinical manifestations (14, 15).

Among the emerging therapeutic options, Sirolimus has stood out as a promising intervention, modulating the immune response and reducing the clinical manifestations of ALPS (16, 17). Previous studies, including systematic reviews have described its effectiveness in improving patients’ quality of life, as well as its ability to induce remission in refractory conditions to conventional therapy (18).

The diagnosis of ALPS remains challenging due to its heterogeneous clinical presentation, variable penetrance, and the overlap of symptoms with other lymphoproliferative and autoimmune disorders (2, 19–21). This study aimed to add a piece in a comprehension of a complex disease, providing a clinical, immunophenotypic, and genetic assessment of a family with multiple members affected by ALPS. Here, we present a unique and comprehensive investigation of a large Brazilian family with ALPS. Among the nine individuals studied, four of them met both the clinical and molecular criteria for the disease, evidencing the importance of familial screening in identifying affected individuals who might otherwise remain undiagnosed. By analyzing these relatives, we aimed to characterize the phenotypic variability of ALPS, explore its incomplete penetrance, and identify potential genotype-phenotype correlations, thereby contributing to a deeper understanding of the disease and its diagnostic challenges. This family-based study highlights the value of integrating multimodal diagnostic approaches to enhance the detection and management of ALPS cases.

## 2 Case description

This study was conducted at Brasília Children's Hospital (Brazil) and was approved by the local Research Ethical Committee (FEPECS-2.332.383). Informed consent was obtained from the patient's caregivers and from adult relatives.

An 11-year-old boy, who was born from consanguineous Brazilian parents, at 38 weeks gestational age, by cesarean section, in good conditions with an Apgar score of 9 and birth weight of 2,675 g. His medical history was negative for recurrent infections, and he had normal psychomotor development. His mother had hypothyroidism. His father was diagnosed with splenomegaly, although no other clinical features, as autoimmune cytopenia, was identified until the end of data collecting. The index patient has two siblings, one asymptomatic boy from his father's side and one girl from her mother's side. His sister has attention deficit disorder and intellectual disability. The patient also had a positive family history of autoimmune diseases, including psoriasis, autoimmune thyroiditis and benign nodules in mammary glands. However, no family history of pediatric cancer was described.

At the age of 3 years and 7 months, the patient was referred to Brasília Children's Hospital, where he began follow-up with our team, after presenting with a three-day fever and a cutaneous rash on the face and scalp, initially treated with oral cephalexin. Despite antibiotic treatment, after a couple of days his condition worsened, and he presented with asthenia and pallor. The patient presented with a clinical feature of cardiac decompensation secondary to severe anemia, as well as abdominal pain. He was then hospitalized for diagnostic elucidation. He received blood transfusion, but with persistence of fever, in association with neutropenia, intravenous antibiotic was prescribed. During that time, tests for infectious diseases were performed, including HIV, viral Hepatitis, Toxoplasmosis, Leishmaniasis, Parvovirus, and others, which were negative.

The patient condition progressed to multilineage cytopenia (immune neutropenia and autoimmune hemolytic anemia) and he was sent to pediatric oncology service. Bone marrow aspirate ruled out hematological malignancies. Oral corticosteroid was prescribed due to a presumed diagnosis of autoimmune hemolytic anemia, and he was discharged after 12 days of hospitalization. The patient had partial response to corticosteroids, and he was sent to an immunologic evaluation, due to high levels of vitamin B12 (>2,000 pg/mL) in association with cytopenia and benign lymphoproliferative disease with suspicion of ALPS diagnosis.

## 3 Diagnostic assessment headings

### 3.1 Clinical and laboratory evaluation

Clinical and laboratory data were collected from medical records. At the first immunological evaluation at 3 years and 8 months of age, the proband exhibited normal growth and development, with no dysmorphic features. However, physical examination revealed splenomegaly and cervical lymphadenopathy. Baseline immunological assessments showed increased immunoglobulin serum levels, a normal vaccine response to rubella and measles as well as normal levels of complement protein. Autoimmune screening showed negative antinuclear and antiphospholipid antibodies, but a positive direct Coombs test was detected. A previous abdominal ultrasound performed at the age of 3 years and 7 months had already evidenced lymphadenopathy and splenomegaly (9 cm).

### 3.2 Immunophenotyping

To further characterize the immunological profile of the patient and some family members, immunophenotyping was performed. Peripheral blood samples were processed using standard flow cytometry protocols. A comprehensive panel of monoclonal antibodies targeting B and T cell markers (CD19, IgD, IgM, CD38, CD27, CD3, CD4, CD8, CD45RA and CCR7) was used to characterize lymphocyte subsets, with data acquisition performed on a BD FACSCanto™ II cytometer and analyzed using FlowJo™ FACS Diva™ software. Notably, the patient presented with reduced CD4+ T cell counts and an increased percentage of circulating CD3 + TCRαβ+CD4−CD8− double-negative T (DNT) cells (10.8%).

A segregation study was performed and his father had elevated double-negative T (DNT) cells (6.9%), low IgM levels, and anemia, with no clinical manifestations of lymphoproliferation. He was sent to a clinical immunologist and an abdominal ultrasound evidenced splenomegaly. The patient's parents are first cousins, and given the consanguinity, a comprehensive familial investigation was initiated. The study included eight additional paternal relatives: the mother, father, half-brother (paternal), grandmother (paternal), two great-aunts, one paternal uncle, and one paternal aunt.

### 3.3 Genetic sequencing

The patient fulfilled the clinical criteria for ALPS, and the diagnosis was confirmed by Sanger sequencing, which identified the pathogenic variant *FAS*:c.748C > T (p.Arg250Ter) in heterozygosity. Segregation analysis demonstrated that this variant was inherited from the patient's father.

To gain deeper insights into the genetic basis of the disease and to validate the immunophenotypic findings, next-generation sequencing (NGS) was performed using a targeted panel covering part of the genes associated with inborn errors of immunity, representing a subset of those listed by the 2024 IUIS classification (Supplementary Table 1). NGS was conducted on the proband and his parents, identifying variants related to ALPS in the *FAS*, *CASP10*, and *LRBA* genes. These variants were subsequently confirmed by polymerase chain reaction (PCR) with gene-specific primers, followed by Sanger sequencing. Additionally, Sanger sequencing of these variants was performed for all family members included in the study. Electropherogram analyses were conducted using CLC Workbench Software v6.4 (QIAGEN).

### 3.4 Functional apoptosis assay

A functional apoptosis assay was performed to evaluate FAS-mediated apoptosis in the proband, his father (both with variants in *FAS* and *CASP10*), and his half-brother (*FAS* variant only). PBMCs were isolated via Ficoll gradient, cultured in RPMI-1640 with supplements, and incubated under standard conditions following the protocol provided in the datasheet of the CD95 antibody (BD Biosciences, Cat# 555670). Cells were exposed to control, rProtG, CD95 + rProtG, and monastrol (positive control). After incubation, samples were stained with antibodies (CD3, CD4, CD8, CD27, CD45, CD45RA, TCR*γδ*, CD5) and analyzed by flow cytometry to assess apoptosis in T effector memory and double-negative T cell subsets.

## 4 Therapeutic intervention

**Figure 1:**
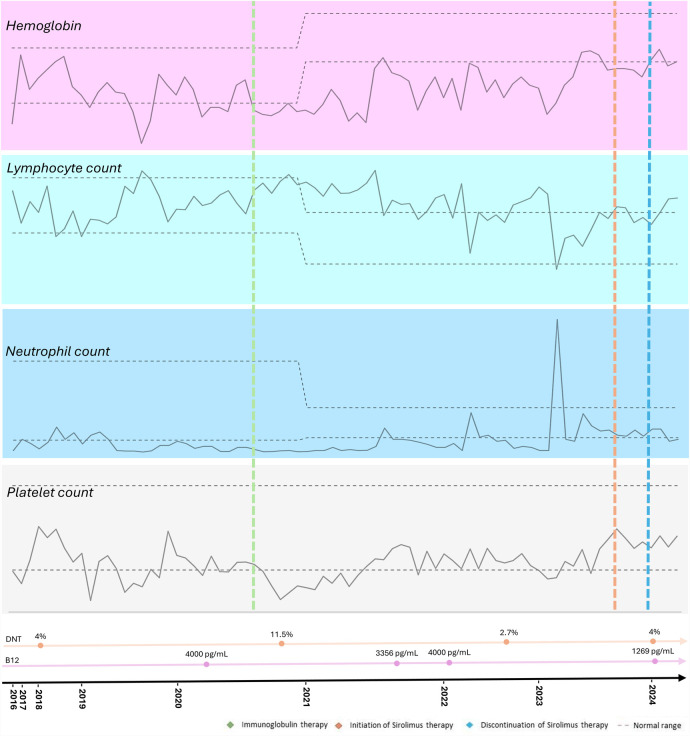
Timeline of the proband's hematological parameters from diagnosis of autoimmune lymphoproliferative syndrome (ALPS) to the present, highlighting key clinical events, treatments, and their impact on blood cell counts.

The therapeutic intervention is summarized in Figure 1. The proband received standard treatment with corticosteroids and initially demonstrated a favorable clinical response. However, due to relapses, regular intravenous immunoglobulin (IVIG) therapy was prescribed, at an immunomodulatory starting dose of 25 g, that was adjusted according to the patient's weight. No adverse events were reported. The patient is currently receiving a reduced replacement dose of immunoglobulin (0.6 g/kg/month), following an initial immunomodulatory regimen, with the aim of managing associated immunodeficiency and reducing infection risk, in association with Sirolimus. Sirolimus was introduced in May 2023, with clinical improvement, and no relapses was noted since then. The starting dose was 2 mg/m^2^/day from November 2023 to March 2024, but unfortunately it was stopped due to difficulties to get the medication from the Brazilian health department. The medication was prescribed again in April 2024 at the same starting dose.

## 5 Follow-up and outcomes

The patient has been followed for seven years and he was hospitalized twice during this period, with a total length of stay of 20 days. The cause of hospital admissions was febrile neutropenia associated with oral thrush. Intravenous antibiotics were used with good outcomes. Apart from that, he was also treated with oral antibiotics 12 times because of upper respiratory tract infections. The most common antibiotics used were Azithromycin and Amoxicillin-clavulanate.

Relapses of cytopenia were noted but with response to oral corticosteroids, and he also had fluctuating splenomegaly. Since the introduction of sirolimus at the age of 9 years, no abnormal images suggestive of active lymphoproliferative disease or cytopenia in the complete blood count (CBC) has been observed. He is still under IVIG therapy in association with sirolimus with good adherence. No adverse events to the therapy have been noted. The proband is currently undergoing a reduction in the dose of immunoglobulin due to the use of sirolimus, showing improvements in blood cell counts, decreased lymphoproliferation, without requiring high doses of corticosteroids (Figure 1).

**Figure 2:**
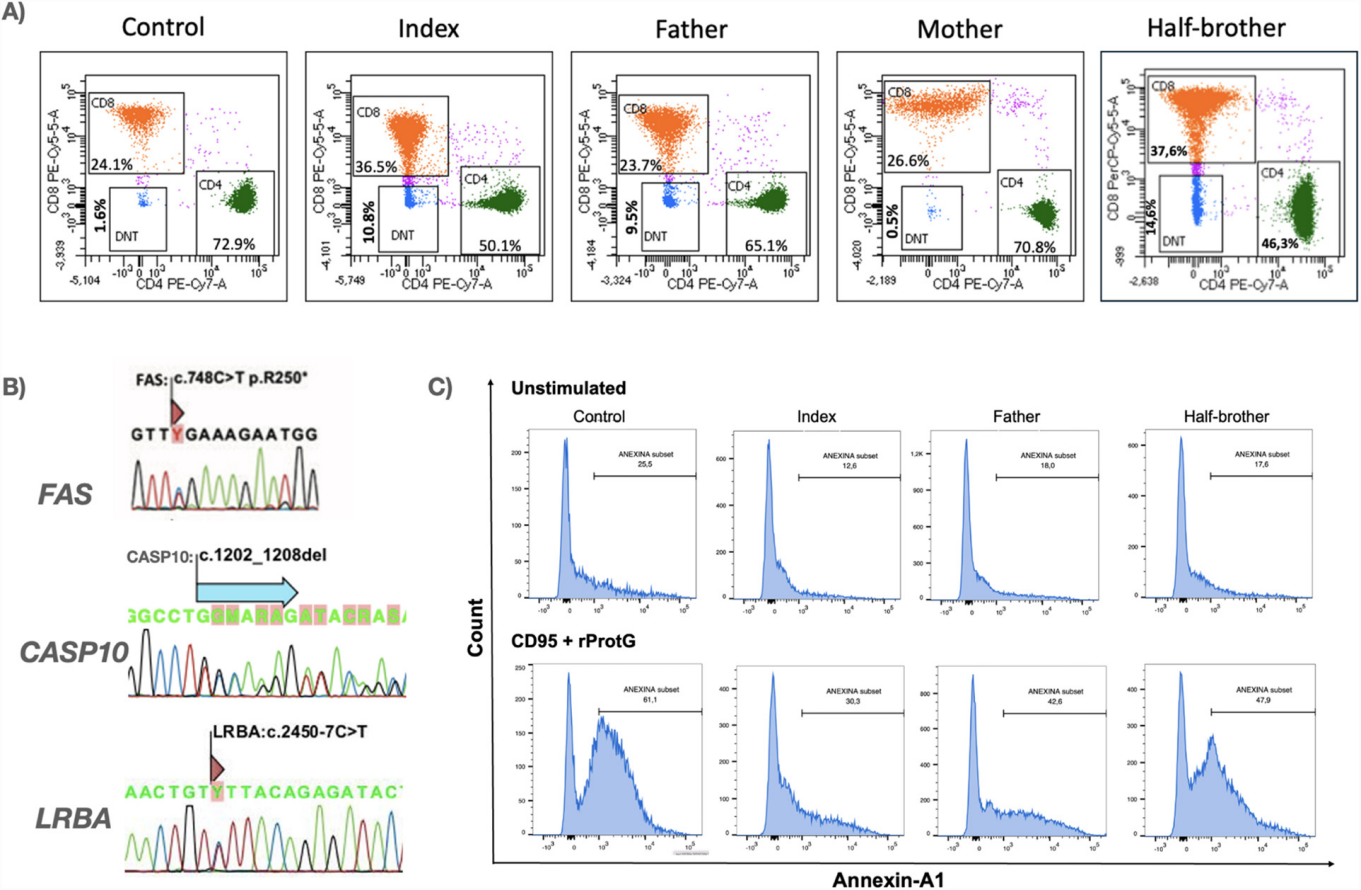
Immunophenotypic, molecular, and functional characterization of the proband and his family. **(A)** Immunophenotyping of circulating lymphocytes demonstrating elevated percentages of CD3 + TCRα/β+CD4-CD8- double-negative T (DNT) cells in the index case and affected family members. Total lymphocyte counts at the time of immunophenotyping were as follows: index—994/μL, father—1,100/μL, mother—3,072/μL, and half-brother—1,942/μL. **(B)** Genetic analysis of *FAS*, *CASP10*, and *LRBA* by RT-PCR followed by Sanger sequencing. *FAS* variant (*FAS*:c.748C > T) was identified in all paternal relatives, while a deletion in *CASP10:*c.1202_1208del) was detected in three paternal family members. The index case and his mother carried a *LRBA* variant (*LRBA:*c.2450-7C > T). **(C)** Functional apoptosis assay evaluating Fas-mediated apoptotic responses in peripheral blood mononuclear cells (PBMCs). Cells from the index case, relatives, and a healthy donor were stimulated with agonistic anti-Fas antibody CD95 (5 μg/100 μL) plus rProtG (1 μg/100 μL) for 21 hours. Apoptosis was assessed via Annexin V staining and flow cytometric analysis of CD45, CD45RA, CD3, CD4, CD8, CD27, and TCRγ/δ markers, revealing defects in apoptotic signaling.

The patient, his father and his half-brother exhibited elevated double-negative T (DNT) cells, whereas the mother had DNT levels within the reference range (Figure 2A). Next-generation sequencing (NGS) analysis of the proband identified a pathogenic variant in *FAS*:c.748C > T (p.Arg250Ter), a variant of uncertain significance in *CASP10:*c.1202_1208del:p.(Cys401Leufs*15), both in heterozygosity, with an autosomal dominant inheritance pattern, inherited from the father. Additionally, a variant in *LRBA:*c.2450-7C > T:p.? was detected in heterozygosity, with an autosomal recessive inheritance pattern, inherited from the mother (Figure 2B).

**Figure 3:**
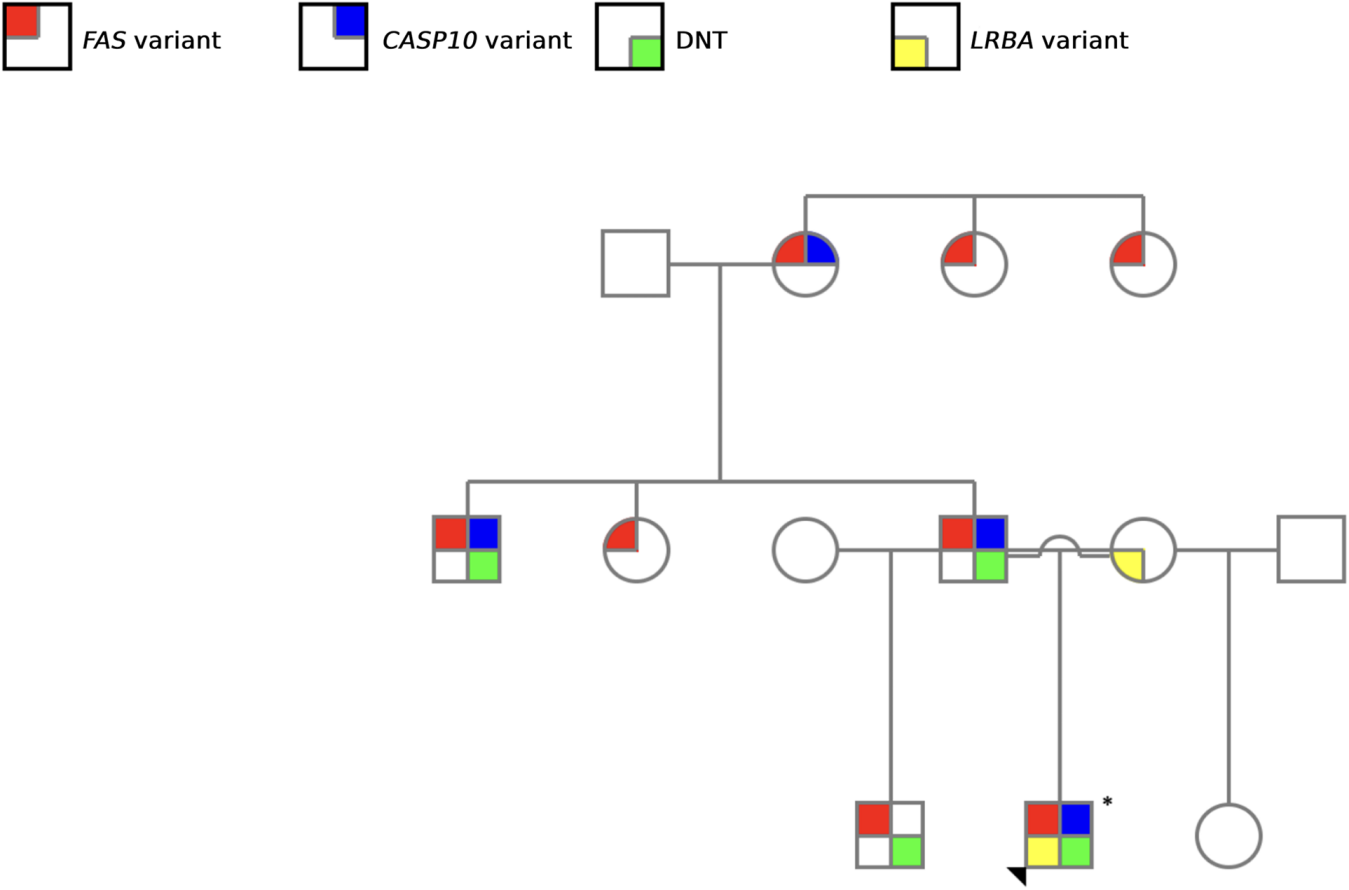
Family pedigree illustrating the screened genetic alterations. Red: *FAS* variant; blue: *CASP10* variant; yellow: *LRBA* variant, and green: elevated DNT cells. The arrowhead indicated the proband.

Sanger sequencing of *FAS*, *CASP10* and *LRBA* was performed for all family members included in the study, revealing the presence of the *FAS* variant in all paternal family members (*n* = 8; 4 males and 4 females). Additionally, a concomitant *CASP10* variant was identified in half of these individuals (*n* = 4; 3 males and 1 female) (Table 1). Correlating molecular findings with clinical presentation and DNT elevation, only male individuals exhibited characteristic symptoms of the disease. Notably, the paternal grandmother, who carried variants in both *FAS* and *CASP10*, remained asymptomatic. Furthermore, disease-associated phenotypic expression varied significantly among male carriers, despite sharing the same variants (Table 1). The proband displayed the most severe clinical manifestations, requiring retherapeutic interventions.

**Table 1:** Clinical and laboratory findings of nine Brazilian patients of the same family according to revised 2009 HIH diagnostic criteria for autoimmune lymphoproliferative syndrome.

Patient information			Required criteria		Accessory criteria				Diagnosis status
					Primary		Secondary		
Sample	Family member	Sex	Chronic (>6 mo), nonmalignant, noninfectious lymphadenopathy or splenomegaly or both	Elevated DNT (>2,5% of CD3+/TCRαβ+)	Defective lymphocyte apoptosis	Somatic or germline pathogenic variants in *FAS, FASL* or *CASP10*	Elevated plasma-soluble FAS ligand levels (>200 pg/mL) OR IL-10 (>20 pg/mL) OR vitamin B12 (>1,500 ng/L) OR IL-18 (>500 pg/mL)	Autoimmune cytopenias and elevated IgG level	
ALPS 15	Index	M	Present	Present	Present	*FAS*/*CASP10*	Present	Present	Confirmed ALPS
ALPS 37	Father	M	Present	Present	Present	*FAS*/*CASP10*	Present	Present	Confirmed ALPS
ALPS 38	Mother	F	Absent	Absent	Absent	Absent	Absent	Absent	Unaffected
ALPS 73	Half-brother	M	Present	Present	Absent	*FAS*	Absent	Absent	Confirmed ALPS
ALPS 74	Grandmother	F	Absent	Absent	Not Evaluated	*FAS*/*CASP10*	Absent	Absent	Unaffected
ALPS 77	Aunt	F	Absent	Absent	Not Evaluated	*FAS*	Absent	Absent	Unaffected
ALPS 78	Uncle	M	Present	Present	Not Evaluated	*FAS*/*CASP10*	Absent	Absent	Confirmed ALPS
ALPS 80	Great aunt	F	Absent	Absent	Not Evaluated	*FAS*	Absent	Absent	Unaffected
ALPS 85	Great aunt	F	Absent	Absent	Not Evaluated	*FAS*	Absent	Absent	Unaffected

Additionally, memory B cell analysis (CD19^+^CD27^+^), revealed reduced absolute counts in four of the nine family members evaluated—namely, the index case (most markedly), his father, paternal uncle, and grandmother. Three of these individuals had a confirmed ALPS diagnosis, while the grandmother remained asymptomatic. In contrast, the half-brother, despite meeting diagnostic criteria for ALPS, showed normal memory B cell levels. This finding is consistent with recent evidence indicating that memory B cell reduction is a common immunophenotypic feature in ALPS patients (22).

The functional apoptosis assay further highlighted this pattern of incomplete penetrance. Both the proband and his father carried variants in *FAS* and *CASP10*, while the half-brother harbored only a *FAS* variant. All three exhibited chronic lymphadenopathy. Notably, the apoptotic activity levels varied among them, with the proband exhibiting the most impaired apoptosis, followed by his father and then his half-brother (Figure 2C).

## 6 Discussion

Autoimmune Lymphoproliferative Syndrome (ALPS) is a rare and often underdiagnosed disorder with less than several hundred cases reported globally (8). In Brazil, the number of documented cases remains scarce, with few studies focusing on this condition (23). Since 2020, scientific literature has advanced in understanding the mechanisms underlying this condition, revealing intricate interactions between genetic, environmental, and immunological factors that favor its manifestation, but the multifaceted clinical presentation of ALPS requires further understanding (24, 25).

Our study is particularly relevant as it encompasses nine members of the same family, allowing the diagnosis of four ALPS patients based on clinical and molecular criteria (Figure 3). Notably, aside from the proband, all other affected family members were only diagnosed as a result of the comprehensive evaluation undertaken in this study. This highlights the challenges associated with ALPS diagnosis, which arise from the heterogeneity of its clinical manifestations—both in terms of symptom presentation and severity—as well as the complexity of establishing a definitive diagnosis (2, 26).

The variable phenotypic expression observed within the family highlight the importance of molecular and functional assays in addition to clinical criteria. It allows to identify asymptomatic or oligosymptomatic individuals who might otherwise remain undiagnosed. This emphasizes the importance of greater awareness and routine family screening in suspected cases, which can support earlier diagnosis, better patient management, and timely interventions.

This family-based analysis reinforces key characteristics of ALPS previously described in the literature, such as incomplete penetrance and a male predominance in clinical manifestations (27–29). ALPS has a higher prevalence in males and epidemiological studies suggest a possible interaction between genetic and hormonal factors that favor the manifestation of the disease in this group (16, 30). In our study, only male individuals exhibited clinical signs of the disease, and a striking example was a female family member who, despite carrying both *FAS* and *CASP10* variants, remained asymptomatic.

Although the *FAS* variant identified in this family is located within the death domain, typically associated with autosomal dominant ALPS, our findings illustrate incomplete clinical penetrance, as not all carriers developed symptoms. This variability suggests that, beyond the location and type of variant (31), additional genetic, epigenetic, or environmental factors likely modulate disease expression. The differing clinical presentations among individuals with identical *FAS* and *CASP10* variants underscore the complexity of genotype-phenotype correlations in ALPS and reinforce the need for further studies to elucidate the mechanisms influencing penetrance and disease severity.

Furthermore, the functional apoptosis assay provided additional support for the diagnosis, confirming the pathogenic role of the identified variants. Our findings suggest that additional variants, such as those in *CASP10* and in *LRBA*, may play a key role in modulating apoptosis in ALPS. The *CASP10* variant identified in our study *CASP10:*c.1202_1208del:p.(Cys401Leufs*15) has been previously reported and functionally evaluated, showing reduced protein expression without impairing FAS-mediated apoptosis (10). These findings support the notion that this alteration alone is unlikely to cause ALPS. However, considering the co-occurrence of this variant with a pathogenic *FAS* variant and the known influence of additional genetic factors on disease penetrance, its potential modifier role cannot be excluded (12, 19).

Although not directly involved in apoptosis like *FAS* or *CASP10*, *LRBA* dysfunction may contribute to immune dysregulation and phenotypic variability. The *LRBA* variant found in this family, despite being of uncertain significance, may act as a modifier. As LRBA regulates CTLA4 trafficking, its deficiency has been linked to autoimmunity and lymphoproliferation (32, 33), which could help explain the clinical heterogeneity among carriers of the same *FAS* variant.

The identification and diagnosis of ALPS are critical not only for the treatment of autoimmune manifestations and lymphoproliferation but also for long-term surveillance due to the significantly increased risk of malignancies, particularly lymphomas. It is already known that individuals with heterozygous germline *FAS* variants have high risk for both non-Hodgkin and Hodgkin lymphomas, with incidences 14 and 51 times greater than the general population, respectively (34, 35). Thus, early diagnosis including clinical evaluation, molecular tests, and functional assays, is crucial for patients survival. Moreover, the persistence of defective FAS-mediated apoptosis in both lymphoid cells and tumor tissues evidences the role of impaired apoptotic pathways as a key factor in lymphomagenesis, justifying the clinical follow-up of asymptomatic carriers of FAS variants (35, 36).

Standard therapeutic management of ALPS includes corticosteroids and immunosuppressive drugs. However, in refractory cases, new approaches have focused on the use of mTOR inhibitors, such as sirolimus. The therapeutic course of the proband underscores the challenges of managing relapsing autoimmune cytopenias and lymphoproliferative disease in ALPS. Initial treatment with corticosteroids provided temporary benefit, but frequent relapses require use of regular IVIG and, later, sirolimus, which led to a sustained remission. This case supports sirolimus as an effective treatment option for ALPS, consistent with previous reports (18).

Clinical studies have shown the efficacy of sirolimus in ALPS, particularly in patients with ALPS-FAS variants, as observed in the proband. A systematic review in India reported that more than half of the 94 ALPS patients evaluated exhibited a fast, complete, and sustained response to sirolimus, with resolution of lymphoproliferation and cytopenias (18). Additionally, sirolimus has a more favorable safety profile compared to corticosteroids and IVIG, which were previously used in the proband's treatment. Given the risks associated with prolonged corticosteroid use—such as metabolic complications, hypertension, and immunosuppression—and the temporary nature of symptom relief provided by immunoglobulin, sirolimus represents a targeted and less toxic alternative (18).

This study highlights the value of integrating genetic testing and immunophenotyping into the diagnostic approach for ALPS, particularly given its clinical and genetic heterogeneity. Identifying asymptomatic carriers supports the need for long-term monitoring, as these individuals may develop symptoms or transmit the disease. Early diagnosis allows timely interventions, potentially reducing complications. Advances in targeted therapies and a better understanding of genotype-phenotype correlations are key to refining diagnostic criteria and improving patient care.

The patient and his family have been actively engaged in the therapeutic approach, and since sirolimus treatment was initiated, no severe relapses of autoimmune cytopenia have been observed. Consequently, this has led to an overall improvement in their quality of life.

## Data Availability

The original contributions presented in the study are included in the article/Supplementary Material, further inquiries can be directed to the corresponding author.

## References

[1] Rao VK Pittaluga S Uzel G. Beyond FAScinating: advances in diagnosis and management of autoimmune lymphoproliferative syndrome and activated PI3 kinase δ syndrome. Hematology Am Soc Hematol Educ Program. (2024) 2024(1):126–36. 10.1182/hematology.2024000537 39644063 PMC11665610

[2] Xu X Denton J Wu Y Liu J Guan Q Dawson DB Genetic testing in patients with autoimmune lymphoproliferative syndrome: experience of 802 patients at cincinnati children’s hospital medical center. J Clin Immunol. (2024) 44(7):166. 10.1007/s10875-024-01772-z 39060684 PMC11282156

[3] Bleesing JJ. Autoimmune lymphoproliferative syndrome (ALPS): Epidemiology and pathogenesis - UpToDate. (2023).

[4] Consonni F Gambineri E Favre C. ALPS, FAS, and beyond: from inborn errors of immunity to acquired immunodeficiencies. Ann Hematol. (2022) 101(3):469–84. 10.1007/s00277-022-04761-7 35059842 PMC8810460

[5] Pellé O Moreno S Lorenz MR Riller Q Fuehrer M Stolzenberg MC Combined germline and somatic human FADD mutations cause autoimmune lymphoproliferative syndrome. J Allergy Clin Immunol. (2024) 153(1):203–15. 10.1016/j.jaci.2023.09.028 37793571

[6] Kuehn HS Niemela JE Rangel-Santos A Zhang M Pittaluga S Stoddard JL Loss-of-function of the protein kinase C δ (PKCδ) causes a B-cell lymphoproliferative syndrome in humans. Blood. (2013) 121(16):3117–25. 10.1182/blood-2012-12-469544 23430113 PMC3630827

[7] López-Nevado M Docampo-Cordeiro J Ramos JT Rodríguez-Pena R Gil-López C Sánchez-Ramón S Next generation sequencing for detecting somatic fas mutations in patients with autoimmune lymphoproliferative syndrome. Front Immunol. (2021) 12:656356. 10.3389/fimmu.2021.656356 33995372 PMC8117005

[8] Hafezi N Zaki-Dizaji M Nirouei M Asadi G Sharifinejad N Jamee M Clinical, immunological, and genetic features in 780 patients with autoimmune lymphoproliferative syndrome (ALPS) and ALPS-like diseases: a systematic review. Pediatr Allergy Immunol. (2021) 32(7):1519–32. 10.1111/pai.13535 33963613

[9] Rieux-Laucat F Magérus-Chatinet A Neven B. The autoimmune lymphoproliferative syndrome with defective FAS or FAS-ligand functions. J Clin Immunol. (2018) 38(5):558–68. 10.1007/s10875-018-0523-x 29911256

[10] Consonni F Moreno S Vinuales Colell B Stolzenberg MC Fernandes A Parisot M Study of the potential role of CASPASE-10 mutations in the development of autoimmune lymphoproliferative syndrome. Cell Death Dis. (2024) 15(5):315. 10.1038/s41419-024-06679-6 38704374 PMC11069523

[11] Miano M Cappelli E Pezzulla A Venè R Grossi A Terranova P FAS-mediated apoptosis impairment in patients with ALPS/ALPS-like phenotype carrying variants on CASP10 gene. Br J Haematol. (2019) 187(4):502–8. 10.1111/bjh.16098 31309545

[12] Zhu S Hsu AP Vacek MM Zheng L Schäffer AA Dale JK Genetic alterations in caspase-10 may be causative or protective in autoimmune lymphoproliferative syndrome. Hum Genet. (2006) 119(3):284–94. 10.1007/s00439-006-0138-9 16446975

[13] Delgadillo DM Céspedes-Cruz AI Ríos-Castro E Rodríguez Maldonado MG López-Nogueda M Márquez-Gutiérrez M Differential expression of proteins in an atypical presentation of autoimmune lymphoproliferative syndrome. Int J Mol Sci. (2022) 23(10):5366. 10.3390/ijms23105366 35628184 PMC9140392

[14] Palmisani E Miano M Grossi A Lanciotti M Lupia M Terranova P Autoimmune lymphoproliferative syndrome (ALPS) disease and ALPS phenotype: are they two distinct entities?. Hemasphere. (2023) 7(3):e845. 10.1097/HS9.0000000000000845 36844186 PMC9949771

[15] Suárez-Gutiérrez M Yamazaki-Nakashimada MA Espinosa S Blancas-Galicia L Lugo-Reyes SO Álvarez-Cardona A. Autoimmune lymphoproliferative syndrome. Update and review. Rev Alerg Mex. (2019) 66(4):456–73. 10.29262/ram.v66i4.646 32105427

[16] Bride K Teachey D. Autoimmune lymphoproliferative syndrome: more than a FAScinating disease. F1000Res. (2017) 6:1928. 10.12688/f1000research.11545.1 29123652 PMC5668920

[17] Gu H Chen Z Ma J Fu L Zhang R Wang T Case report: effectiveness of sirolimus in a d*e novoF*AS mutation leading to autoimmune lymphoproliferative syndrome-FAS and elevated DNT/treg ratio. Front Pediatr. (2022) 10:868193. 10.3389/fped.2022.868193 35967554 PMC9366043

[18] Sharma S Hussain MS Agarwal NB Bhurani D Khan MA Ansari MAA. Efficacy of sirolimus for treatment of autoimmune lymphoproliferative syndrome: a systematic review of open label clinical studies. Expert Opin Orphan Drugs. (2021) 9(7–10):219–26. 10.1080/21678707.2021.1970523

[19] Wang J Zheng L Lobito A Chan FK Dale J Sneller M Inherited human caspase 10 mutations underlie defective lymphocyte and dendritic cell apoptosis in autoimmune lymphoproliferative syndrome type II. Cell. (1999) 98(1):47–58. 10.1016/S0092-8674(00)80605-4 10412980

[20] Chun HJ Zheng L Ahmad M Wang J Speirs CK Siegel RM Pleiotropic defects in lymphocyte activation caused by caspase-8 mutations lead to human immunodeficiency. Nature. (2002) 419(6905):395–9. 10.1038/nature01063 12353035

[21] Neven B Magerus-Chatinet A Florkin B Gobert D Lambotte O De Somer L A survey of 90 patients with autoimmune lymphoproliferative syndrome related to TNFRSF6 mutation. Blood. (2011) 118(18):4798–807. 10.1182/blood-2011-04-347641 21885602

[22] Staniek J Kalina T Andrieux G Boerries M Janowska I Fuentes M Non-apoptotic FAS signaling controls mTOR activation and extrafollicular maturation in human B cells. Sci Immunol. (2024) 9(91):eadj5948. 10.1126/sciimmunol.adj5948 38215192

[23] Kanegane H Vilela MM Wang Y Futatani T Matsukura H Miyawaki T. Autoimmune lymphoproliferative syndrome presenting with glomerulonephritis. Pediatr Nephrol. (2003) 18(5):454–6. 10.1007/s00467-003-1087-3 12736807

[24] Matson DR Yang DT. Autoimmune lymphoproliferative syndrome: an overview. Arch Pathol Lab Med. (2020) 144(2):245–51. 10.5858/arpa.2018-0190-RS 30958694 PMC10415410

[25] Rao VK Oliveira JB. How I treat autoimmune lymphoproliferative syndrome. Blood. (2011) 118(22):5741–51. 10.1182/blood-2011-07-325217 21885601 PMC3228494

[26] Tarbox JA Keppel MP Topcagic N Mackin C Ben Abdallah M Baszis KW Elevated double negative T cells in pediatric autoimmunity. J Clin Immunol. (2014) 34(5):594–9. 10.1007/s10875-014-0038-z 24760111 PMC4047151

[27] Adam MP Feldman J Mirzaa GM Pagon RA Wallace SE Amemiya A. GeneReviews. (1993).

[28] Hsu AP Dowdell KC Davis J Niemela JE Anderson SM Shaw PA Autoimmune lymphoproliferative syndrome due to FAS mutations outside the signal-transducing death domain: molecular mechanisms and clinical penetrance. Genet Med. (2012) 14(1):81–9. 10.1038/gim.0b013e3182310b7d 22237435

[29] Jackson CE Fischer RE Hsu AP Anderson SM Choi Y Wang J Autoimmune lymphoproliferative syndrome with defective fas: genotype influences penetrance. Am J Hum Genet. (1999) 64(4):1002–14. 10.1086/302333 10090885 PMC1377824

[30] Paskiewicz A Niu J Chang C. Autoimmune lymphoproliferative syndrome: a disorder of immune dysregulation. Autoimmun Rev. (2023) 22(11):103442. 10.1016/j.autrev.2023.103442 37683818

[31] Le Deist F Emile JF Rieux-Laucat F Benkerrou M Roberts I Brousse N Clinical, immunological, and pathological consequences of fas-deficient conditions. Lancet. (1996) 348(9029):719–23. 10.1016/S0140-6736(96)02293-3 8806292

[32] Lo B Zhang K Lu W Zheng L Zhang Q Kanellopoulou C AUTOIMMUNE DISEASE. Patients with LRBA deficiency show CTLA4 loss and immune dysregulation responsive to Abatacept therapy. Science. (2015) 349(6246):436–40. 10.1126/science.aaa1663 26206937

[33] Martínez Jaramillo C Trujillo-Vargas CM. LRBA in the endomembrane system. Colomb Med (Cali). (2018) 49(3):236–43. 10.25100/cm.v49i3.3802 30410199 PMC6220489

[34] Poppema S Maggio E van den Berg A. Development of lymphoma in autoimmune lymphoproliferative syndrome (ALPS) and its relationship to fas gene mutations. Leuk Lymphoma. (2004) 45(3):423–31. 10.1080/10428190310001593166 15160902

[35] Straus SE Jaffe ES Puck JM Dale JK Elkon KB Rösen-Wolff A The development of lymphomas in families with autoimmune lymphoproliferative syndrome with germline fas mutations and defective lymphocyte apoptosis. Blood. (2001) 98(1):194–200. 10.1182/blood.V98.1.194 11418480

[36] Straus SE Sneller M Lenardo MJ Puck JM Strober W. An inherited disorder of lymphocyte apoptosis: the autoimmune lymphoproliferative syndrome. Ann Intern Med. (1999) 130(7):591–601. 10.7326/0003-4819-130-7-199904060-00020 10189330

