# Supplementary material for: Case Report: Clinical, molecular, and functional characterization of autoimmune lymphoproliferative syndrome—a family study with a multimodal diagnosis
Source: Front Pediatr. 2025 Jul 18;13:1639749. doi: 10.3389/fped.2025.1639749 (PMC12313618; doi:10.3389/fped.2025.1639749)
Supplement: Supplementary file 1 [file Table1.docx]

**Supplemental Table 1**. List of genes included in the Next-Generation Sequencing (NGS) panel used in the molecular investigation of patients with suspected Autoimmune Lymphoproliferative Syndrome (ALPS) and related autoinflammatory or immunodeficiency disorders. The panel targets genes involved in immune regulation, apoptosis, lymphocyte development, and immune signaling pathways.

| **Genes** | | | |
| --- | --- | --- | --- |
| *AICDA* | *AIRE* | *CARD11* | *CARD9* |
| *CASP10* | *CASP8* | *CD19* | *CD27* |
| *CD81* | *CR2* | *DOCK8* | *FADD* |
| *FAS* | *FASLG* | *FERMT1* | *G6PD* |
| *GATA2* | *HAX1* | *ICOS* | *IKZF1* |
| *IL2RA* | *ITCH* | *ITK* | *LAMTOR2* |
| *LRBA* | *LYST* | *MAGT1* | *MALT1* |
| *MS4A1* | *MSH6* | *NFKB2* | *PEPD* |
| *PIK3CD* | *PIK3R1* | *PRF1* | *PTEN* |
| *RASGRP1* | *SH2D1A* | *SLC7A7* | *STAT3* |
| *STK4* | *STX11* | *STXBP2* | *TFRC* |
| *TNFRSF13B* | *TNFRSF13C* | *TNFRSF9* | *UNC13D* |
| *UNG* | *WAS* | *XIAP* |  |
